# BETWEEN THE DEVIL AND THE DEEP BLUE SEA: DISPERSANTS IN THE GULF OF MEXICO

**DOI:** 10.1289/ehp.118-a338

**Published:** 2010-08

**Authors:** Charles W. Schmidt

**Affiliations:** **Charles W. Schmidt**, MS, an award-winning science writer from Portland, ME, has written for *Discover Magazine*, *Science*, and *Nature Medicine*

As this article goes to press, the scope of the worst oil spill in U.S. history remains a moving target. The explosion and collapse of the BP-owned *Deepwater Horizon* oil rig on 20 April 2010 uncorked an underwater geyser that for 85 consecutive days shot an estimated daily load of 1.47–2.52 million gallons^1^—and possibly more—into the Gulf of Mexico. Caused by igniting gases leaking from API Well No. 60-817-44169, located 42 miles off the Louisiana coast and 5,000 feet underwater, the explosion killed 11 workers. At press time more than 600 miles of coastline was fouled by the oil, and about one-third of the Gulf’s fishing grounds were closed.^2^ BP engineers stanched the flow on July 15 with a mechanical cap, but oil and methane seeps have since appeared near the wellhead, raising new questions about the integrity of the well and, indeed, of the seafloor.^2^ No one knows what will happen next.

The *Deepwater Horizon* spill has generated heart-wrenching scenes of dying birds, oil-fouled marshes and barrier islands, and traumatized coastal residents. But a key image from this story isn’t even visible: mysterious plumes of dispersed oil droplets flowing deep underwater. To some degree, these plumes arose from intense physical pressures at the mile-deep wellhead, which broke the oil into droplets that never reached the surface. But spill response workers also used chemical dispersants—mixtures of solvents, surfactants, and other proprietary additives—to achieve a similar effect. Sprayed from the air and applied directly at the gushing wellhead, dispersants changed the oil’s physical and chemical properties, splitting it into tiny droplets that measure roughly 10 microns in diameter (naturally dispersed oil droplets are about 10 times larger).^3^ Dispersed oil droplets get pulled (or “entrained”) into the water column, where they undergo a range of removal processes, mainly metabolism by marine bacteria.

The decision to use dispersants—which have been commercially available for oil spill response since the mid-1960s—always involves environmental tradeoffs, says Mahlon Kennicutt, a professor of chemical oceanography at Texas A&M University. Whereas undispersed oil floats on water, smothering birds and marine mammals and fouling coastal resources, dispersed oil is transported throughout the water column, where it’s more available to marine life. “Dispersants don’t make the oil go away,” Kennicutt emphasizes.

“Zooplankton mistake oil droplets for food,” adds Carys Mitchelmore, an associate professor at the University of Maryland’s Chesapeake Biological Laboratory. That’s a dangerous scenario because zooplankton are crucial to the marine food web. Kill them off, Mitchelmore says, and the consequences spiral upward.

## Difficult Decision

As of this writing, nearly 2 million gallons of dispersants have been applied to the oil in a deliberate effort to protect the Gulf’s ecologically sensitive coastlines.^2^ Still, a number of prominent environmentalists have questioned the wisdom of this use, given how little scientists know about their ecologic impacts, particularly in the deep sea. In a May 24 blog post, Richard Denison, a senior scientist with the Environmental Defense Fund, wrote that “the unanswered questions, data gaps, and withheld [confidential business] information surrounding BP’s use of dispersants are flowing in seemingly as fast as the oil is leaking.”^4^

Denison and others have questioned whether it wouldn’t be better to leave the oil undispersed. Mitchelmore explains that, when trapped underwater, oil’s lighter, more volatile components—namely, aromatic compounds that include benzene,^5^ toluene,^6^ ethylbenzene,^7^ and xylene^8^ (BTEX)—can’t evaporate into the air. Instead, they remain in the water, where they pose an acute threat to undersea life, says Ronald Tjeerdema, chairman of the Department of Environmental Toxicology at the University of California, Davis. But Tjeerdema points out that although BTEX compounds are acutely toxic, they aren’t persistent—they are rapidly broken down in the ocean, and they don’t bioaccumulate in fish tissue.

Carl Safina, a marine ecologist and president of the Blue Ocean Institute, an environmental organization in Cold Spring Harbor, New York, puts it another way: “Dispersants just allow the oil to reach a much greater expanse of the marine environment.”

Yet during a meeting sponsored by the National Oceanic and Atmospheric Administration (NOAA) and the U.S. Environmental Protection Agency (EPA), held May 26–27 at Louisiana State University, more than 50 scientists concluded that dispersant applications to that point had been appropriate for the *Deepwater Horizon* response.^9^ Booms, skimmers, controlled burns, and other mechanical tools for controlling oil spills on the surface are certainly preferable to using chemicals in the ocean, acknowledges Nancy Kinner, co-director of the University of New Hampshire Coastal Response Research Center, which administered the meeting. But surface recovery rarely exceeds 10% from any oil spill, she says, and mechanical recovery only works in calm weather.

“Even without hurricanes, you get a lot of wind and waves this time of the year in the Gulf,” Kinner says. “And when we looked at oil getting into coastal wetlands and affecting the species that go through them, we quickly realized that dispersants had to be seen as a major response tool. It was our conclusion that both the use of dispersants and the effects of dispersed oil in the water column are generally less harmful than allowing the oil to be on the surface so it migrates to nearshore habitat.” The scientists also recommended that dispersant effects be reevaluated on an ongoing basis, Kinner emphasizes, to ensure their use remains justified.

Mitchelmore says officials might have a hard time discerning when dispersants are doing more harm than good. “The sheer volume of dispersants over this extended time period and the depths we’re applying them are novel,” she says. “We’re in unchartered territory.”

In a report published by the National Research Council in 2005,^3^ scientists acknowledged that much of what happens to chemically dispersed oil at sea remains a mystery. The rate at which it binds to sediments is unknown, as is how quickly it breaks down in the ocean, how it’s ingested and taken up by undersea organisms, and what sorts of by-products are created when microbes degrade it.

According to a spokeswoman with the EPA press office, the agency “reserves the right to stop the application of dispersant if any negative impacts on the environment outweigh the benefits.” Those impacts, she explains, could be assessed using two approaches: by measuring dissolved oxygen (DO) in Gulf water samples and by testing for mortality among small marine invertebrates called rotifers when exposed to Gulf water. According to the EPA, the Gulf’s normal DO concentration is 4 mg/L.^10^ But as aerobic microbes metabolize organic compounds, including oil, they consume and remove oxygen from water, and that can stress marine organisms if DO concentrations fall too low.

If DO levels drop below 2 mg/L, the EPA spokeswoman says, then the ongoing use of dispersants would be called into question. Mitchelmore cautions these thresholds may not be so definitive, however, given that scientists know little about deep-sea life forms in the Gulf and their dependence on specific DO levels. “Without knowing more about the organisms living there, you can’t make the call about DO effects,” Mitchelmore says.

## Corexit Takes the Spotlight

Before the *Deepwater Horizon* explosion, “dispersant” was hardly a household word. But when vast amounts of the chemicals were suddenly used in the Gulf this year, the public demanded more information on them. At first, reporters and even other scientists didn’t have much to go on. The two products used in the spill, Corexit® 9500 and 9527, are manufactured by Nalco, a company based in Sugar Land, Texas. The Material Safety Data Sheet (MSDS) for each dispersant indicates they contain one of two solvents: 2-butoxyethanol (2-BE), found in Corexit 9527^11^—an older product dating back to the 1970s—or petroleum distillates, found in the newer Corexit 9500^12^ product. The Corexit dispersants also contain organic sulfonic acid salt (a surfactant) and propylene glycol (a stabilizer).

The MSDSs also made reference to undisclosed proprietary components, and that raised immediate suspicion—newspapers warned that “secret formulations” were being dumped into Gulf waters. Alex Madonik, a chemistry consultant with the Green Science Policy Institute, a toxics-reduction think tank in Berkeley, California, explains that Nalco had no legal obligation to publicly disclose proprietary ingredients in its dispersants and that the EPA—which was privy to that information—had agreed to keep it confidential. But as public demands for transparency grew, the agency posted a full list of Corexit ingredients on its website in mid-June.^10^ Among the undisclosed ingredients were several surfactants, including sorbitan and 1-(2-butoxy-1-methylethoxy) 2-propanol, which according to Madonik is a solvent/antifreeze mixture. Madonik downplays any potential toxicity from these ingredients, but acknowledges that “some of the compound percentages are stated in broad ranges, and that leaves uncertainty about dose and exposure.”

Citing results mainly from animal studies, the MSDS for Corexit 9527 claims 2-BE may cause hemolysis (destruction of red blood cells) or kidney or liver damage with repeated or excessive exposure, whereas the formulated dispersant also may cause skin or gastrointestinal irritation.^11^ The MSDS also states that “human red blood cells [exposed to 2-BE] have been shown to be significantly less sensitive to hemolysis than those of rodents and rabbits. These effects are transient and when exposure is discontinued, these effects subside.”^11^ In contrast to the MSDS for Corexit 9527, which assigns a “moderate” human health risk to this compound,^11^ the 9500 MSDS lists the human health risk as “slight.”^12^

Corexit 9500 is identical to Corexit 9527 with the sole exception that 2-BE is replaced by petroleum distillates akin to kerosene. According to Tjeerdema, petroleum distillates have less acute toxicity than 2-BE, but as hydrocarbons, they’re more likely to bioaccumulate in marine life. Yet compared with the amounts of hydrocarbon leaking to the Gulf from the oil spill, “what’s added by Corexit 9500 is insignificant,” he says.

BP had already stockpiled several hundred thousand gallons of Corexit 9527, which was used in the *Deepwater Horizon* response until supplies ran out in mid-May, according to the EPA.^13^ Since then, the sole dispersant used in the BP cleanup has been Corexit 9500.^13^ But weeks after use of Corexit 9527 was supposedly phased out, BP released data indicating 2-BE was still being detected in 20% of personal air samples collected offshore^14^—a finding that has not been explained. Frank Mirer, a professor of toxicology at Hunter College, comments, “It is implausible that this fraction of samples with detectable levels would be found if 2-BE were no longer being used. This calls into question the accuracy of dispersant usage information being reported.”

## Toxicity: The Big Question

It’s difficult to isolate the human health effects of dispersants given that dispersant and crude oil exposures happen simultaneously. Cleanup workers have complained of headache, shortness of breath, dizziness, and nausea, and it’s possible that dispersant exposure aggravates these effects, in part by enabling oil to more easily penetrate the skin, according to Kathy Burns, a toxicologist and director of Sciencecorps, a coalition of environmental health professionals based in Lexington, Massachusetts. Yet rolling seas, heat, and the overwhelming sensory experience of working in the oil spill also can contribute to these symptoms.

Don Aurand, vice president and senior scientist at Ecosystem Management & Associates, Inc., an environmental consulting firm under contract to BP, says dispersants used today are less toxic than their predecessors. During the world’s first major oil spill, caused when the supertanker *Torrey Canyon* leaked 24–35 million gallons of oil after striking a reef off Great Britain in 1967, workers used chemical degreasers, industrial detergents, kerosene, and other products to disperse the slick.^15^ Those products—never intended for use in oil spills—turned out to be ecologically devastating, Aurand says. In contrast, he says, newer dispersants designed specifically for oil spill response balance maximal effectiveness at breaking up a slick with minimal toxicity on their own. How they achieve that balance can’t easily be assessed, however, because the formulations—being trade secrets—are protected against public disclosure.

The EPA’s National Contingency Plan Product Schedule^16^ shows the Corexit products to be near the bottom of the list of approved dispersants in terms of effectiveness. But Gina Coelho, president of Ecosystem Management & Associates, claims the agency’s efficacy testing methods were flawed. “Several decades of research by industry, academia, and spill response organizations show the Corexit products are the dispersants of choice; they work better on weathered and fresh oil, and you can use them over a range of temperatures,” she says.

As far as toxicity goes, a good deal of concern focuses around the effects not of the dispersants alone but in combination with crude oil. Dana Wetzel, a senior scientist and program manager at Mote Marine Laboratory who studies the effects of dispersants on marine life, says that compared with oil’s water-soluble fractions (i.e., the BTEX components), the Corexit products are three orders of magnitude less harmful to marine test organisms including *Mysidopsis bahia*, *Menidia beryllina*, and *Sciaenops ocellatus*. Wetzel based her findings on a comparison of acute LC_50_ values (which reflect the “lethal concentration” required to kill 50% of test organisms) obtained in her laboratory.^17^ However, LC_50_ values for dispersed oil were the same as for oil’s water-soluble fractions, Wetzel says. She adds that neither she nor her colleagues in the field have adequately explored dispersants’ sublethal effects, “so we don’t know how the organisms’ dispersant burdens or body burdens for dispersed oil relate to changes in reproduction or immune function.”

Mitchelmore points out that LC_50_ values for the same dispersant or dispersed oil mixtures vary widely among different species, and even among different life stages within species: “In the scientific literature you can see orders-of-magnitude differences in Corexit toxicity depending on species and life stages.” For instance, she says, among the 13 approved dispersants listed on the EPA’s National Contingency Plan Product Schedule,^18^ Corexit 9500 mixed with number 2 fuel oil is listed as the most toxic to fish, but it’s the sixth most toxic to shrimp.

Meanwhile, toxicologists interviewed for this article unanimously say LC_50_ values have questionable ecologic relevance. “They don’t mimic real-life exposure,” Coelho says. “LC_50_ tests generally last twenty-four to forty-eight hours at sustained concentrations much higher than what we actually see in the environment.” Yvonne Addassi, a senior environmental scientist with the California Department of Fish and Game’s Office of Spill Prevention and Response, says better results can be obtained with “spiked/declining” exposure tests, during which peak concentrations fall over time to mimic the effects of tidal cycles.

Nevertheless, the EPA relies chiefly on LC_50_ data for dispersant comparisons, and on May 20, the agency directed BP to choose a different dispersant with LC_50_ values either greater than (indicating less toxicity) or equal to 23 ppm for *Menidia* and 18 ppm for *Mysidopsis*^19^ (although Mitchelmore points out the directive mistakenly said “less than or equal to”). These values—as reflected in the agency’s National Contingency Plan Product Schedule,^18^ which is now being revised—correspond to Sea Brat #4, a dispersant made by Alabaster Corporation. The EPA’s directive was immediately rejected by BP, however, which claimed Sea Brat #4 metabolizes to an endocrine-disrupting chemical, nonylphenol, that could persist in the environment for years.^20^ Neither of the Corexit products degrades to nonylphenol, BP officials countered, and each of them biodegrades completely in the ocean within 28 days.

Under pressure to verify the accuracy of the LC_50_ data, the EPA reassessed eight approved dispersants and found that Corexit 9500 was only “slightly toxic” to the aquatic invertebrate *Americamysis bahia* and “practically nontoxic” to *M. beryllina*.^21^ In reassessment results published separately, Richard S. Judson and colleagues report that none of the dispersants tested displayed significant endocrine-disrupting activity.^22^ (Corexit 9527 was not tested.) The EPA is now examining the acute toxicity of Louisiana sweet crude oil alone and in combination with dispersants.

## Real-Time Experiment

Since the spill, multiple research vessels have been conducting sampling in the Gulf. David L. Jones, a research associate at the University of South Florida, was aboard the university’s research vessel (R/V) *Weatherbird II*, which reported sampling results gathered May 22–28 at three stations, two of them at 40 and 45 nautical miles northeast and southeast of the wellhead, respectively, and another 142 miles southwest of the wellhead. NOAA’s analysis of the *Weatherbird II*’s data revealed hydrocarbon concentrations in the range of less than 0.5 ppm while polyaromatic hydrocarbon (PAH) levels measured in the parts per trillion.^23^ Moreover, rotifer toxicity test results gathered by scientists on the R/V *Brooks McCall*, leased to BP, had yet to detect organism mortality in any sample above 20%, according to Coelho, who coordinates the *Brooks McCall*’s research activities.

But David Valentine, a professor of microbial geochemistry at the University of California, Santa Barbara, says he collected samples June 11–20 while aboard the R/V *Cape Hatteras* that may be cause for concern. Valentine observed oxygen declines of 5–35% within plumes at 2,500 feet or deeper. These plumes were located within a 5- to 7-mile radius of the spill site, where hydrocarbon levels ranged from 10,000 to 100,000 times over background.

“We just have no idea how big the subsea plume is,” Jones says. “It’s possible we’re just tracking subsea surface slicks that could be the tip of an iceberg.”

In a blog posting from June 20, Samantha Joye, a professor of marine biology at the University of Georgia, who was in the Gulf aboard the R/V *Pelican* and the R/V *Walton Smith* May 25–June 6, claimed her data revealed a southwest plume extending more than 20 miles from the spill site and a northeast plume traceable to 30 miles.^24^ Joye says she will post “general” results on her blog but that more precise PAH measurements would be held back pending publication in a peer-reviewed journal. “I can’t [release actual data plots to the media] because that would render them unpublishable,” she says. “Rest assured that the actual data plots are being provided in real time to the responders—NOAA, EPA, etc.—and we’re doing all we can to get the data out ASAP in publication form.”

In June 9 testimony to the House Subcommittee on Energy and Environment, Joye emphasized that because oil dispersed underwater can’t be cleaned up, it has the potential to influence oceanic ecosystems for years.^25^ Its fate lies almost entirely with metabolizing bacteria, which could promote hypoxic or anoxic conditions in the deep ocean. “Deep water [oxygen] isn’t replenished *in situ* by photosynthesis,” she says. “Rather, it’s replaced by physical processes”—that is, waters “turn over” (rise to the surface) every few decades. If the deep Gulf becomes anoxic, she speculates, microbes could switch to sulfate reduction (instead of aerobic metabolism, which relies on oxygen), raising the potential for substantial volumes of anoxic, sulfidic water.

Joye says these impacts could spread steadily eastward toward the West Florida shelf, a band of rock and coral reefs running along the coast at depths of roughly 160–400 feet. This Marine Protected Area contains crucial habitat for some of the Gulf’s most ecologically and commercially important fish species, such as grouper, Jones says. “Plankton at the base of the food chain could be affected, and you could see heightened toxicity among younger species in general,” he says. “If a mass of groupers aggregates to spawn in one contaminated area, the adults might survive while the hatchlings could die.”

But Joye admits that the farther scientists get from the spill site, the harder it will be for them to link hydrocarbon pollution to the spill versus natural seeps that release an estimated 24–61 million gallons of oil to the Gulf every year.^26^ The Gulf is often described as having a “leaky” seafloor, where some biological communities have adapted to metabolize oil and constrain its impacts, Joye says. But although these creatures can tolerate hydrocarbons and low DO levels, the “impact of the BP blowout will challenge their tolerance . . . beyond any previous insult,” she says.

## Not the First Time

Among the more remarkable stories emerging from this disaster is that advances in deepwater drilling haven’t been matched by progress on spill response. Indeed, responses to the *Deepwater Horizon* disaster are essentially the same as those applied during another massive spill in the Gulf more than 30 years ago: booms, skimmers, dispersants, failed “capping” attempts, and finally new relief wells. Caused by an exploding oilrig operated by PEMEX (Mexico’s national oil company) in roughly 300 feet of water 50 miles northwest of Ciudad del Carmen, that spill released more than 146 million gallons of oil before relief wells were drilled nine months later.^27^ More than 2.5 million gallons of dispersant (about 75% of which was Corexit products) were used in the spill, according to Olof Lindén, a professor at the World Maritime University, who coauthored a 1981 research article on the PEMEX spill.^27^

“I’m surprised there’s no reasonable solution except the drilling of new relief wells—after all, you would expect, after thirty years, some progress in the development of alternative techniques to collect oil from spills from the seabed,” says Lindén. In contrast to the rampant pessimism surrounding the current spill, Lindén strikes a more optimistic note, noting that unlike Alaska’s Prince William Sound—where the *Exxon Valdez* ran aground in 1989—the Gulf has a warmer, subtropical climate that accelerates the degradation of the oil.

“You have enormous dilution potential in the open waters of the Gulf,” Lindén says. “I don’t think this is the end of the Gulf of Mexico or of the productivity of coastal waters off Louisiana, Mississippi, and Alabama. If you ask me, the more serious problems here are overfishing and the release of organic nutrients [carried southward in the Mississippi River] that cause local oxygen depletion.”

Still, studies worldwide show that oil spills generate coastal impacts that can last many decades. Unlike oil at sea, which is metabolized fairly quickly by aerobic bacteria, oil-polluted beaches and marshes rely chiefly on anaerobic degradation, which is painfully slow by comparison. Below just 10–15 cm in beach sand and 2–3 cm in muddier sediments, oxygen levels plummet, according to Markus Heuttel, a professor of biological oceanography at Florida State University who is currently studying this phenomenon. And from these anoxic layers, pockets of oil can leach toxicants for decades, he says. It could take that long for scientists to fully grasp the environmental consequences of this disaster.

## Figures and Tables

**Figure:**
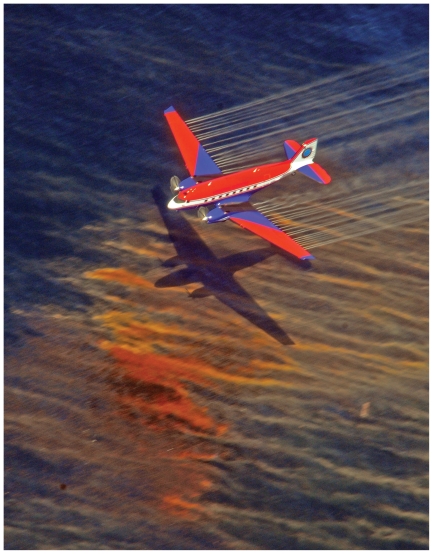

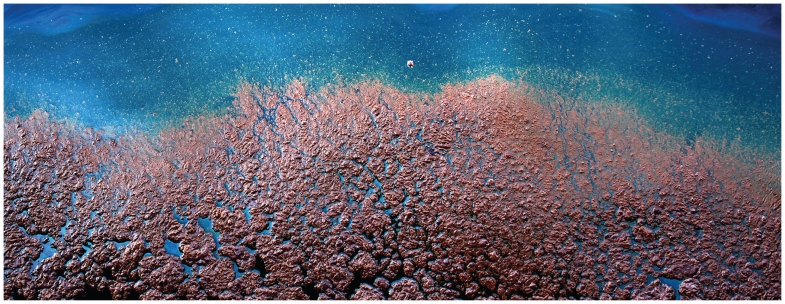
Dispersant is released off the coast of Houma, Louisiana, from a Basler BT-67 fixed-wing aircraft, 5 May 2010 (above). Surface oil (right, photographed 7 June 2010 off the coast of Pensacola, Florida) will—with wind, wave action, and other factors—naturally disperse to some degree. The addition of chemical dispersants enhances the process, allowing a large part of the surface slick to move into the water column in the form of tiny droplets. At press time, nearly 2 million gallons of dispersants have been applied to the Gulf of Mexico. About 42% of that has been applied subsea at depths where these chemicals have never been tested.

**Figure:**
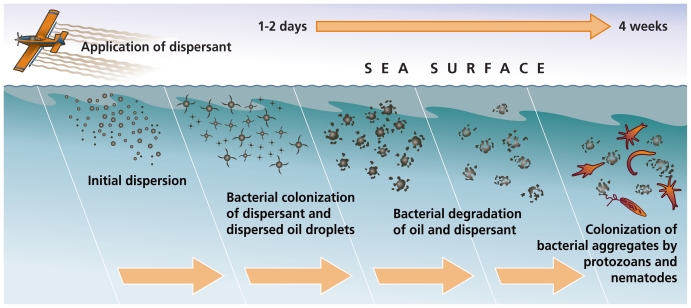
**The decision to apply dispersants is time sensitive—dispersants are usually most effective if applied within the first 48 hours of a spill.** Adapted from: Clark J. Dispersant Basics: Mechanism, Chemistry, and Physics of Dispersants in Oil Spill Response. Presentation to NRC Committee on Understanding Oil Spill Dispersants: Efficacy and Effects, 15 March 2004.

**Figure:**
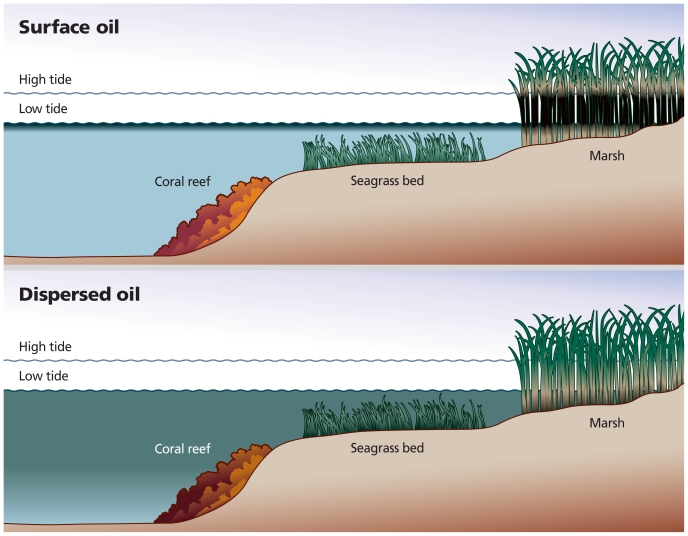
**The use of dispersants always involves an environmental tradeoff. Floating oil will not affect coral or seagrass, but it can devastate coastlines. Dispersed oil, on the other hand, becomes far more available to underwater organisms but largely spares coastal ecosystems.** Adapted from: Clark J. Dispersant Basics: Mechanism, Chemistry, and Physics of Dispersants in Oil Spill Response. Presentation to NRC Committee on Understanding Oil Spill Dispersants: Efficacy and Effects, 15 March 2004.

**Figure:**
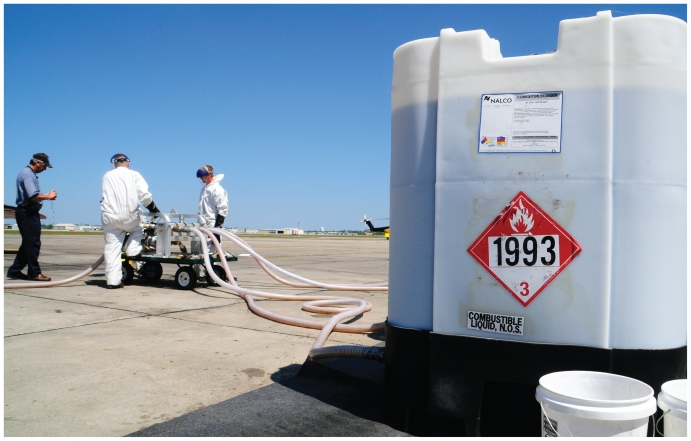
Corexit dispersant is pumped from tanks in preparation for aerial application, Houma, Louisiana, 5 May 2010.
